# Acute ingestion of a ketone monoester, whey protein, or their co-ingestion in the overnight postabsorptive state elicit a similar stimulation of myofibrillar protein synthesis rates in young males: a double-blind randomized trial

**DOI:** 10.1016/j.ajcnut.2024.01.004

**Published:** 2024-01-11

**Authors:** Sarkis J Hannaian, Jamie Lov, Stephanie E Hawley, Manon Dargegen, Divine Malenda, Ari Gritsas, Gilles Gouspillou, José A Morais, Tyler A Churchward-Venne

**Affiliations:** 1 Department of Kinesiology and Physical Education, McGill University, Montréal, Quebec, Canada; 2 Research Institute of the McGill University Health Centre, Montréal, Quebec, Canada; 3 Département des Sciences de l’activité Physique, Faculté des Sciences, UQAM, Montréal, Quebec, Canada; 4 Division of Geriatric Medicine, McGill University, Montréal, Quebec, Canada

**Keywords:** ketone bodies, young males, myofibrillar protein synthesis, β-hydroxybutyrate, whey protein, exogenous ketosis, skeletal muscle, amino acids

## Abstract

**Background:**

Ketone bodies may have anabolic effects in skeletal muscle via their capacity to stimulate protein synthesis. Whether orally ingested exogenous ketones can stimulate postprandial myofibrillar protein synthesis (MyoPS) rates with and without dietary protein co-ingestion is unknown.

**Objectives:**

This study aimed to evaluate the effects of ketone monoester intake and elevated blood β-hydroxybutyrate (β-OHB) concentration, with and without dietary protein co-ingestion, on postprandial MyoPS rates and mechanistic target of rapamycin complex 1 (mTORC1) pathway signaling.

**Methods:**

In a randomized, double-blind, parallel group design, 36 recreationally active healthy young males (age: 24.2 ± 4.1 y; body fat: 20.9% ± 5.8%; body mass index: 23.4 ± 2 kg/m^2^) received a primed continuous infusion of L-[*ring*-^2^H_5_]-phenylalanine and ingested one of the following: *1*) the ketone monoester (R)-3-hydroxybutyl (R)-3-hydroxybutyrate (KET), *2*) 10 g whey protein (PRO), or *3*) the combination of both (KET+PRO). Blood and muscle biopsy samples were collected during basal and postprandial (300 min) conditions to assess β-OHB, glucose, insulin, and amino acid concentrations, MyoPS rates, and mTORC1 pathway signaling.

**Results:**

Capillary blood β-OHB concentration increased similarly during postprandial conditions in KET and KET+PRO, with both being greater than PRO from 30 to 180 min (treatment × time interaction: *P* < 0.001). Postprandial plasma leucine and essential amino acid (EAA) incremental area under the curve (iAUC) over 300 min was greater (treatment: both *P* < 0.001) in KET+PRO compared with PRO and KET. KET, PRO, and KET+PRO stimulated postprandial MyoPS rates (0–300 min) higher than basal conditions [absolute change: 0.020%/h; (95% CI: 0.013, 0.027%/h), 0.014%/h (95% CI: 0.009, 0.019%/h), 0.019%/h (95% CI: 0.014, 0.024%/h), respectively (time: *P* < 0.001)], with no difference between treatments (treatment: *P* = 0.383) or treatment × time interaction (interaction: *P* = 0.245). mTORC1 pathway signaling responses did not differ between treatments (all *P* > 0.05).

**Conclusions:**

Acute oral intake of a ketone monoester, 10 g whey protein, or their co-ingestion in the overnight postabsorptive state elicit a similar stimulation of postprandial MyoPS rates in healthy young males.

This trial was registered at clinicaltrials.gov as NCT04565444 (https://clinicaltrials.gov/study/NCT04565444).

## Introduction

Ketone bodies [ie, β-hydroxybutyrate (β-OHB), acetoacetate, and acetone] are naturally occurring lipid-derived molecules whose endogenous production is amplified in the liver through ketogenesis in response to low energy availability (ie, prolonged fasting, and starvation), very low carbohydrate intake (ie, a ketogenic diet), and prolonged glycogen depleting exercise [1]. Under these conditions, ketone bodies serve as a metabolic fuel source in extrahepatic tissues, such as the brain, heart, and skeletal muscles [1]. In addition to serving as a fuel source, β-OHB (the primary ketone body in circulation) is now recognized as a signaling metabolite that can modulate an array of physiologic functions, such as substrate metabolism, inflammation, oxidative stress, and gene expression in multiple organs including skeletal muscle [2]. Recently, orally ingested exogenous ketone supplements have been developed that can rapidly (within minutes) induce a transient (for ∼3 h) hyperketonemia (blood β-OHB ∼3–5 mM) without the need for dietary alteration [3,4]. Therefore, exogenous ketone supplements permit direct testing of the metabolic effects of elevated blood ketone body concentration without the confounding influence of widespread changes that occur in response to prolonged fasting/starvation or ketogenic diets.

The role of ketone bodies in the regulation of whole-body and muscle protein metabolism has been a topic of interest for decades. Early studies in humans reported that intravenous infusion of ketone bodies reduced urinary nitrogen excretion by >30% during prolonged fasting [5,6], suggesting that ketone bodies may have protein sparing effects. In skeletal muscle, intravenous infusion of sodium DL-β-OHB has been reported to stimulate muscle protein synthesis (MPS) rates in both dogs [7] and humans [8]. More recently, Vandoorne et al. [9] reported that oral intake of an exogenous ketone monoester supplement co-ingested with a protein-carbohydrate–containing beverage enhanced the phosphorylation status of select proteins within the mechanistic target of rapamycin complex 1 (mTORC1) signaling pathway in human skeletal muscle after exercise [9]. In the same study [9], addition of ketone bodies to a low dose of leucine (1.5 mM) stimulated a ∼2-fold increase in protein synthesis in C_2_C_12_ cells, which was similar to that achieved with a high-dose of leucine (5.0 mM). Altogether, these data suggest that orally ingested exogenous ketone supplements may promote protein synthesis in muscle, potentially through activation of the mTORC1 signaling pathway. However, the effects of orally ingested exogenous ketone supplements, with and without dietary protein co-ingestion, on both myofibrillar protein synthesis (MyoPS) rates and mTORC1 pathway signaling have not been evaluated.

The aim of this study was to evaluate the effects of elevated blood β-OHB through oral ingestion of the ketone monoester (R)-3-hydroxybutyl (R)-3-hydroxybutyrate when consumed without protein (KET) and when co-ingested with 10 g whey protein (KET+PRO), on postprandial MyoPS rates and compare with those of protein ingestion (PRO). In addition, we examined the phosphorylation status of select signaling proteins within the mTORC1 pathway known to be involved in the regulation of MyoPS rates. Given the established dose–response relationship between protein/essential amino acid (EAA) ingestion and postprandial MPS rates, whereby ingestion of 20 g high-quality protein [10,11] or 10 g EAA [12] maximally stimulates postprandial MPS rates in young males, we hypothesized that PRO would stimulate MyoPS rates higher than basal conditions (ie, overnight postabsorptive conditions). We further hypothesized that KET would stimulate MyoPS rates higher than basal conditions; however, KET+PRO would stimulate greater MyoPS rates than both KET and PRO alone due to ketone bodies amplifying the anabolic effect of a suboptimal 10 g dose of protein.

## Methods

### Participants and ethical approval

Thirty-six healthy recreationally active young males (age: 24.2 ± 4.1 y) were recruited to participate in this randomized, double-blind, parallel group study. Healthy was characterized as a body mass index >18.5 and <30 kg/m^2^ and moderately active based on responses to a routine screening questionnaire. Participants with an exercise frequency between 3 and 4 times per week were considered recreationally active and were included in the study. Participants with any identified metabolic or intestinal disorders, self-reported use of tobacco products, allergies to milk proteins, lactose intolerance, phenylketonuria, a history of neuromuscular problems, previous participation in a stable-isotope tracer study, adherence to a strict vegan or vegetarian diet, current use of ketone supplements or adherence to a ketogenic diet, use of medications known to affect protein metabolism, diagnosis of diabetes, and participation in sports or physical exercise for ≥5 d per week were excluded from the study. Participants were recruited through advertisements on dedicated bulletin boards within McGill University and posts on social media from 14 September 2020 to 26 April 2021. The intervention/treatment experimental test days were conducted between 21 September 2020, and 14 May 2021. All participants were informed about the purpose of the study, the experimental procedures, and potential risks before providing informed written consent. The study was conducted in accordance with the ethical standards of the Faculty of Medicine Institutional Review Board at McGill University on human experimentation and in accordance with the tenets of the Helsinki Declaration of 1975 as revised in October 2013. The study was approved by the Faculty of Medicine and Health Sciences Institutional Review Board at McGill University on 6 January, 2020 (IRB Study Number: A11-M51-19A) and was registered at clinicaltrials.gov (NCT04565444).

### Preliminary testing, diet, and physical activity

Participants underwent an initial laboratory screening visit to assess height, weight, blood pressure, and body composition (through dual-energy x-ray absorptiometry; GE Healthcare). All participants were provided with food intake and physical activity logs that they were asked to complete during the 2 d immediately before their experimental trial visit. During this time, study participants were asked to refrain from strenuous physical activity and alcohol consumption. This was verified verbally with the participant on the morning of their experimental trial and confirmed by checking their food intake and physical activity logs. Dietary intake from the food logs was analyzed using commercially available software (Food Processor version 11.9; ESHA Research). All participants were provided with a standardized meal (Michelina’s Beef and Macaroni; Bellisio Foods), which provided 2134 kJ of energy and consisted of 52% energy from carbohydrates, 31% fat, and 17% protein. Participants were instructed to store the meal in their freezer and consume it for dinner during the evening before their experimental trial visit. The participants were instructed to stop consuming food or beverages (except water) by 21:00, after which they were instructed to fast until testing the following morning.

### Experimental design

This study used a randomized, double-blind, parallel group design in which participants reported to the laboratory for a single test visit (not including the preliminary testing visit). Participants were randomly assigned to 1 of the 3 nutritional beverage treatment groups (*n* = 12 participants per group). The randomization procedure to allocate treatment group was executed using a random-number generator (www.randomization.com). An investigator not directly affiliated with the study was responsible for the randomization. Beverage specifications are outlined further*.* To limit diurnal and intrasubject variation, all measures were performed according to a standardized time schedule at the same time of day.

### Nutritional treatments

An investigator not directly affiliated with the study was responsible for the preparation of the treatment beverages on the morning of the experimental visit. Treatments were matched for volume and prepared in opaque plastic bottles. KET and KET+PRO treatment groups ingested the ketone monoester (R)-3-hydroxybutyl (R)-3-hydroxybutyrate (DeltaG; TDeltaS; under license by HVMN) at a dose of 0.36 g/kg/body weight per serving. Previous studies have shown a single 0.36g/kg/body weight dose of (R)-3-hydroxybutyl (R)-3-hydroxybutyrate to be safe, tolerable, and effective in eliciting acute hyperketonemia in humans (ie, raising plasma β-OHB to ∼3 mM) [3,4]. Participants in PRO and KET+PRO groups consumed protein beverages containing 10 g of whey protein concentrate (Isagenix). The protein-containing beverages were enriched to 4% with L-[*ring*-^2^H_5_]-phenylalanine, based on a phenylalanine content of 3.14% in the whey protein, to minimize disturbance in the plasma precursor pool enrichments [13]. To better match the energy content of the nutritional treatments, carbohydrate was added to the KET and PRO treatments. This consisted of a combination of Glacier Cherry Gatorade (G2 Gatorade Company), dextrose powder (NOW foods), stevia (Stevia Select), and vanilla flavoring (McCormick & Company). The stevia and vanilla flavoring were added to the nutritional treatments to better match taste. After consumption, the bottle containing the nutritional treatment was rinsed with 40 mL of water to dislodge any remaining remnants and ensure that all content in the bottle was consumed. The participants had 5 min to ingest the nutritional treatment assigned to them. Treatment specifications are outlined in Table 1.

**TABLE 1:** Amino acid, ketone monoester, carbohydrate, fat, and protein content of the nutritional treatments

	Nutritional treatment group		
	KET	PRO	KET+PRO
Amino acid content			
Alanine (g)	—	0.49	0.49
Arginine (g)	—	0.25	0.25
Aspartic acid (g)	—	1.03	1.03
Cysteine (g)	—	0.26	0.26
Glutamic acid (g)	—	1.67	1.67
Glycine (g)	—	0.19	0.19
Histidine (g)	—	0.18	0.18
Isoleucine (g)	—	0.64	0.64
Leucine (g)	—	1.04	1.04
Lysine (g)	—	0.84	0.84
Methionine (g)	—	0.23	0.23
Phenylalanine (g)	—	0.32	0.32
Proline (g)	—	0.59	0.59
Serine (g)	—	0.49	0.49
Threonine (g)	—	0.68	0.68
Tryptophan (g)	—	0.22	0.22
Tyrosine (g)	—	0.32	0.32
Valine (g)	—	0.58	0.58
ΣNEAA (g)	—	4.73	4.73
ΣEAA (g)	—	5.27	5.27
Beverage totals			
Ketone monoester (g)	24.0–29.7	—	18.3–29.7
Carbohydrate (g)	12.2	24.4–36.6	1.1
Fat (g)	—	0.6	0.6
Protein (g)	—	10.0	10.0
Energy (kJ)	586–815	627–844	601–830

ΣEAA, sum total essential amino acids; KET, treatment consisting of 0.36 g/kg/body weight of the ketone monoester (R)-3-hydroxybutyl (R)-3-hydroxybutyrate; KET+PRO, treatment consisting of 0.36 g/kg/body weight of the ketone monoester (R)-3-hydroxybutyl (R)-3-hydroxybutyrate co-ingested with 10 g whey protein; ΣNEAA, sum total nonessential amino acids; PRO, treatment consisting of 10 g whey protein.

### Experimental protocol

**FIGURE 1:**
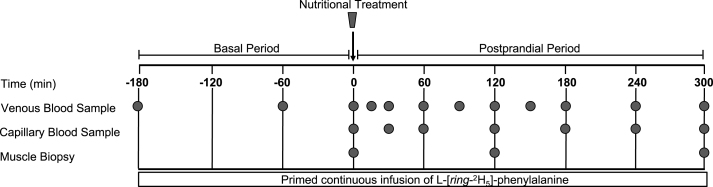
Schematic of the experimental protocol. Participants were randomly assigned to consume 1 of the 3 nutritional treatments (KET, PRO, or KET+PRO; *n* = 12 in each group). A single basal (ie, overnight postabsorptive) biopsy was collected at *t* = 0 min, followed by the ingestion of the nutritional treatment. Filled circles represent venous blood, capillary blood, and muscle biopsy samples at the respective time points.

A schematic overview of the experimental protocol is shown in Figure 1. Study participants reported to the laboratory at ∼07:30 in the ∼10-h overnight postabsorptive state. On arrival, participants rested comfortably on a bed in the laboratory while health status and compliance of the pre-experimental visit guidelines were confirmed. A Polytetrafluoroethylene catheter was inserted into an antecubital vein for baseline blood sample collection (*t* = −180 min) and for the infusion of L-[*ring*-^2^H_5_]-phenylalanine. The stable-isotope tracer L-[*ring*-^2^H_5_]-phenylalanine (ACP Chemicals) was dissolved in 0.9% saline for intravenous infusion. The tracer solution was prepared at a pharmacy (Gentès & Bolduc, Pharmaciens) and subsequently tested for sterility. After the administration of a priming dose (2.2 μmol/kg) of the L-[*ring*-^2^H_5_]-phenylalanine tracer, a calibrated syringe pump (Harvard Apparatus) was used to continuously infuse the tracer during the entire experimental trial (8 h, 0.05 μmol/kg/min). A second Polytetrafluoroethylene catheter was inserted into the dorsal hand vein or antecubital vein of the contralateral arm for heated (60 °C) arterialized venous blood sampling. A saline drip was connected to the stopcock to keep the catheter patent for repeated blood sampling. Another arterialized venous blood sample was drawn at *t* = −60 min. Before the nutritional treatment, a muscle biopsy was collected along with another arterialized venous blood sample (*t* = 0 min) to determine basal (ie, overnight postabsorptive) MyoPS rates and mTORC1-related signaling molecule phosphorylation status. Immediately after the skeletal muscle biopsy and arterialized venous blood sample at *t* = 0 min, a capillary blood sample was obtained through finger lancet to measure basal β-OHB concentration. Then, participants ingested 1 of the 3 nutritional treatments based on treatment group randomization (ie, PRO, KET, and KET+PRO). Arterialized venous blood samples (8 mL each) were subsequently collected at *t* = 15, 30, 60, 90, 120, 150, 180, 240, and 300 min during the postprandial period after treatment intake. Arterialized venous blood samples were drawn into a prechilled 8-mL blood collection tube (BD Vacutainer) coated with K2EDTA. All tubes were inverted 10 times and centrifuged at 3000 × *g* for 15 min at 4 °C. After centrifugation, the plasma samples were aliquoted out into microtubes. All plasma samples were frozen in liquid nitrogen and transferred into a −80 °C freezer until further analysis. Capillary blood samples were subsequently collected at *t* = 30, 60, 90, 120, 180, 240, and 300 min to measure β-OHB concentration using a handheld blood ketone monitor (FreeStyle Precision Neo). The capillary samples were collected using a lancet after cleaning with alcohol and allowing the area to air dry. The first blood droplet sample was discarded with a cotton swab, and the subsequent droplet samples were used for analysis. Two additional skeletal muscle biopsies were collected at *t* = 120 min and *t* = 300 min to permit assessment of temporal changes in MyoPS rates and signaling molecule phosphorylation status between the nutritional treatments in the postprandial state. All biopsies were obtained in a distal to proximal manner from the middle region of the vastus lateralis muscle (15 cm above the patella) through separate incisions (2–3 cm apart) ∼2 cm below entry through the fascia with a UCH-style skeletal muscle biopsy needle with suction under local anesthesia (2% Lidocaine; Teligent.) [14,15]. After muscle biopsy, the incision was closed using a Steristrip and covered with a pressure bandage. Muscle samples were separated from any visible blood, adipose, and connective tissue, then immediately flash frozen in liquid nitrogen and stored at −80 °C until subsequent analysis.

### Plasma and muscle tissue analyses

The Supplemental Methods provide details on the preparation and analysis relating to the determination of plasma glucose, insulin, and amino acid concentrations and free and protein-bound L-[*ring*-^2^H_5_]-phenylalanine enrichments in blood and muscle. In brief, ∼30 mg muscle tissue was homogenized using a tissue homogenizer over ice in 2 mL Optima water. The homogenate was centrifugated to separate the myofibrillar and collagen fractions. The myofibrillar pellet was further processed (ie, resolubilized, hydrolyzed, heated, evaporated, and reconstituted) before analysis by ultraperformance liquid chromatography–mass spectrometry.

### Calculations

The fractional synthesis rate (FSR) of myofibrillar proteins was assessed using the following standard precursor–product equation:

$$\text{FSR}\;\left(\%/h\right)=\Delta E_{p}/\left[E_{\text{pl}}\times t\right]\times 100\%$$

where ΔE_p_ is the change in protein-bound L-[*ring*-^2^H_5_]-phenylalanine enrichment between 2 muscle biopsies, E_pl_ is the weighted mean plasma-free L-[*ring*-^2^H_5_]-phenylalanine precursor enrichment in mole percentage excess (MPE) across the 2 biopsy samples, and *t* is the tracer incorporation time in hours. Weighted mean plasma enrichments were calculated by taking the measured enrichment between consecutive time points and correcting for the time between these sampling time points. The use of tracer-naïve participants in this study allowed the use of the preinfusion blood sample (*t* = −180 min; ie, a mixed plasma protein fraction) as the baseline enrichment for the calculation of basal (ie, overnight postabsorptive) FSR. This approach has been previously validated [13,16,17].

### Western blotting

The Supplemental Methods provide details of the analysis relating to the determination of the phosphorylation status of mTORC1-related signaling targets [ie, protein kinase B (Akt^Ser473^), mechanistic target of rapamycin (mTOR^Ser2448^), 70-kDa ribosomal protein S6 kinase (p70S6K^Thr389^), eukaryotic translation initiation factor 4E-binding protein 1 (4E-BP1^Thr37/46^), and ribosomal protein S6 (rpS6^Ser240/244^)]. A list of the antibodies used and their respective dilution and reference numbers for immunoblotting analysis is summarized in Table 2.

**TABLE 2:** List of antibodies and their respective dilution and reference numbers for immunoblotting analysis

Antibody	Dilution	Reference
Phospho-mTOR^Ser2448^	1/500	Cell Signaling Technology #2971
Phospho-Akt^Ser473^	1/500	Cell Signaling Technology #9271
Phospho-p70S6 kinase^Thr389^	1/1,000	Cell Signaling Technology #9205
Phospho-S6 Ribosomal Protein^Ser240/244^	1/1,000	Cell Signaling Technology #2215
Phospho-4E-BP1^Thr37/46^	1/1,000	Cell Signaling Technology #2855

### Statistical analysis

We performed a sample size calculation for a 1-way ANOVA F test to compare postprandial MyoPS rates (over 0–300 min) between 3 treatment groups. We used 0.040%/h as an estimate of the mean postprandial MyoPS rate for both PRO [11] and KET (as we expected them to be similar). We used 0.061%/h as an estimate of the mean postprandial MyoPS rate for KET+PRO, representing a MyoPS rate >50% greater than both PRO and KET. We also estimated the SD of postprandial MyoPS rates across treatment groups to be 0.015%/h [11,18]. Using G∗Power software (version 3.1.9.7) [19,20], we found that with a significance level (α) of 0.05 and an effect size (*f*) of 0.66, a sample size of at least 9 per treatment group would be sufficient to detect a difference in MyoPS rates of 0.021%/h with a power (1 − *β*) of >0.8. However, to account for dropouts, we recruited 12 participants per treatment group.

Plasma glucose, insulin, amino acid, and β-OHB concentration data were assessed using a 2-factor (treatment × time) repeated-measures ANOVA and 1 factor (treatment) ANOVA [for incremental area under the curve (iAUC)]. Plasma-free L-[*ring*-^2^H_5_]-phenylalanine enrichments were assessed using a 2-factor (treatment × time) repeated-measures ANOVA. Skeletal muscle MyoPS rates (ie, FSR) were assessed using a 2-factor (treatment × time) repeated-measures ANOVA. The postprandial time course (ie, 0–120 min and 120–300 min) and aggregate (ie, 0–300 min) FSR were analyzed separately and were compared with the basal postabsorptive (ie, −180 to 0 min) FSR. Bonferroni post hoc analyses were performed when a significant main effect or interaction was observed after the 2-factor repeated-measures ANOVA testing. Assumptions of the statistical models were assessed using Levene test (for 1-factor ANOVA), Mauchley test, and the D’Agostino-Pearson omnibus normality test at a significance of *P* < 0.05. If a significant Mauchley test was determined, the Greenhouse–Geisser correction factor was used to adjust the degrees of freedom accordingly. For data that did not pass normality, values were transformed with the ln or square root of the value. The statistical analysis was performed on transformed data, but nontransformed data are presented in graphic or tabular form for clarity. If a significant Levene test was determined in the 1-factor ANOVA, Welch ANOVA, and the Dunnett T3 post hoc comparison were used accordingly to test for treatment differences. There were no missing data. Statistical analysis was performed with the SPSS, version 26 (IBM) and GraphPad Prism (Prism, version 8; GraphPad Software). In all statistical analysis, statistical significance was set at *P* value of <0.05. Participant characteristics are expressed as mean ± SD. All other data are expressed as mean, 95% CI, and individual participant data where appropriate.

## Results

### Participant characteristics

**FIGURE 2:**
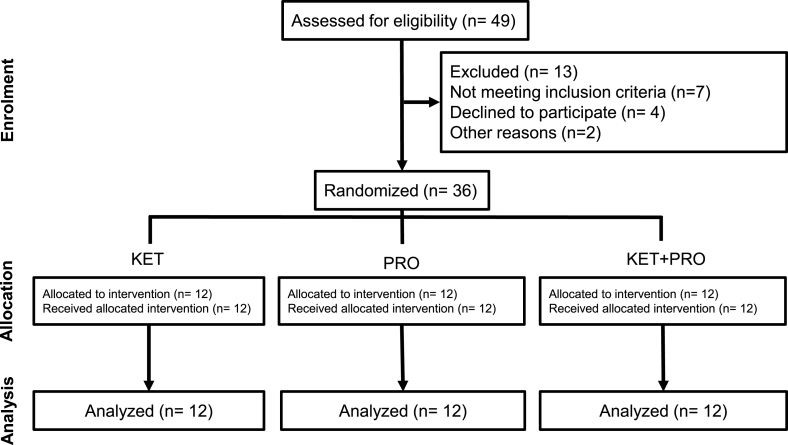
CONSORT flow diagram. CONSORT, Consolidated Standards of Reporting Trials.

Baseline characteristics of the participants who were randomly assigned into the intervention arms and completed the trial are presented in Table 3. Of the 49 participants assessed for eligibility, 13 were excluded (7 did not meet the inclusion criteria, 4 declined to participate, and 2 did not participate for other reasons). Thirty-six participants were randomly assigned to the intervention arms, resulting in *n* = 36 for complete analyses. A CONSORT flow diagram is shown in Figure 2.

**TABLE 3:** Characteristics of male study participants who ingested nutritional treatments consisting of a ketone monoester, whey protein, or ketone monoester co-ingested with whey protein

Characteristic	Nutritional treatment group		
	KET	PRO	KET+PRO
Age (y)	23.3 ± 3.6	24.0 ± 4.7	25.4 ± 3.9
Height (m)	1.75 ± 0.05	1.78 ± 0.07	1.73 ± 0.08
Weight (kg)	74.5 ± 5.2	71.9 ± 8.6	70.0 ± 10.5
BMI (kg/m^2^)	24.3 ± 1.6	22.6 ± 1.7	23.4 ± 2.3
Systolic BP (mm Hg)	121 ± 13	112 ± 10	118 ± 12
Diastolic BP (mm Hg)	77 ± 11	72 ± 10	77 ± 8
Resting heart rate (bpm)	78 ± 13	77 ± 13	73 ± 13
Body fat (%)	22.1 ± 6.5	21.2 ± 4.8	19.6 ± 6.2
Bone-free and fat-free mass (kg)	55.5 ± 5.0	53.8 ± 6.6	53.5 ± 8.2

Values represent mean ± SD, *n* = 12 per group.

Abbreviations: bpm, beats per minute; KET, treatment consisting of 0.36 g/kg/body weight of the ketone monoester (R)-3-hydroxybutyl (R)-3-hydroxybutyrate; KET+PRO, treatment consisting of 0.36 g/kg/body weight of the ketone monoester (R)-3-hydroxybutyl (R)-3-hydroxybutyrate co-ingested with 10 g whey protein; PRO, treatment consisting of 10 g whey protein.

### Dietary intake

Dietary intake data over the 2 d immediately before the experimental visit is tabulated in Table 4. No differences were present between KET, PRO, and KET+PRO treatment groups for total energy (kilojoules), carbohydrate (grams), fat (grams), protein (grams), relative protein intake (grams protein per kilogram of body weight), and percentage of total energy intake as carbohydrate, fat, or protein.

**TABLE 4:** Mean 2-d dietary intake of male study participants who ingested nutritional treatments consisting of a ketone monoester, whey protein, or ketone monoester co-ingested with whey protein

	Nutritional treatment group			*P*
	KET	PRO	KET+PRO	
Energy (kJ/d)	9511 (8258, 10764)	7741 (6527, 8955)	8992 (7497, 10487)	0.18
Carbohydrate (g)	294 (253, 335)	224 (187, 261)	238 (188, 288)	0.08
Fat (g)	81 (69, 93)	74 (54, 94)	86 (64, 108)	0.66
Protein (g)	99 (77, 121)	80 (66, 94)	108 (74, 142)	0.54
Protein (g/kg/d)	1.3 (1.1, 1.5)	1.1 (0.9, 1.3)	1.5 (1.1, 1.9)	0.43
Carbohydrate (% total energy)	52 (48, 56)	49 (43, 55)	46 (37, 55)	0.47
Fat (% total energy)	32 (29, 35)	35 (29, 41)	35 (28, 42)	0.76
Protein (% total energy)	17 (15, 19)	18 (15, 21)	19 (15, 23)	0.78

Values represent means and 95% CI, *n* = 12 per group. Data were analyzed using a 1-factor ANOVA.

KET, treatment consisting of 0.36 g/kg/body weight of the ketone monoester (R)-3-hydroxybutyl (R)-3-hydroxybutyrate; KET+PRO, treatment consisting of 0.36 g/kg/body weight of the ketone monoester (R)-3-hydroxybutyl (R)-3-hydroxybutyrate co-ingested with 10 g whey protein; PRO, treatment consisting of 10 g whey protein.

### Capillary blood β-OHB concentrations

**FIGURE 3:**
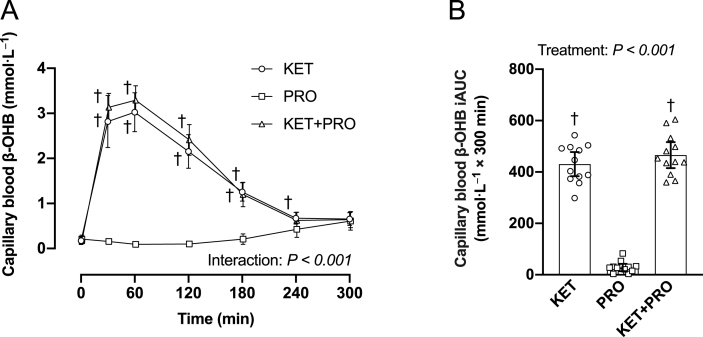
Capillary blood β-OHB (A) concentration (mmol/L) during basal postabsorptive conditions (*t* = 0 min) and during postprandial conditions (*t* = 15–300 min), and corresponding iAUC (B) after beverage intake in young males. Values represent means ± 95% CI and individual participant data, *n* = 12 per treatment. Time-course data were analyzed with a 2-factor repeated-measures ANOVA. Bonferroni post hoc tests were performed to determine the difference between treatments within each time point. iAUC data were analyzed with a 1-factor ANOVA. Bonferroni post hoc tests were performed to detect differences between treatments. If a significant Levene test was determined, Welch ANOVA and the Dunnett T3 post hoc comparison were used accordingly to detect differences between treatments. Time-course data: ^†^a difference from PRO within each time point, *P* < 0.05. The iAUC data: ^†^a difference from PRO, *P* < 0.05. iAUC, incremental area under the curve; KET, treatment consisting of 0.36 g/kg/body weight of the ketone monoester (R)-3-hydroxybutyl (R)-3-hydroxybutyrate; KET+PRO, treatment consisting of 0.36 g/kg/body weight of the ketone monoester (R)-3-hydroxybutyl (R)-3-hydroxybutyrate co-ingested with 10 g whey protein; PRO, treatment consisting of 10 g whey protein.

Capillary blood β-OHB (Figure 3A) concentration (mmol/L) increased (interaction, *P* < 0.001) after ingestion of the ketone monoester and was significantly greater in both KET and KET+PRO from 30 to 180 min in the postprandial period. At 240 min, capillary blood β-OHB concentration was significantly greater in KET (*P* = 0.039) than in PRO. Peak capillary blood β-OHB concentration (C_max_) was greater (*P* < 0.001) in KET (3.2 mmol/L; 95% CI: 2.8, 3.6 mmol/L) and KET+PRO (3.4 mmol/L; 95% CI: 3.1, 3.7 mmol/L) compared with PRO (0.6 mmol/L; 95% CI: 0.4, 0.8 mmol/L), but were not different from each other (*P* = 0.951). The iAUC for capillary blood β-OHB (Figure 3B) over the 300-min postprandial period was greater (*P* < 0.001) in both KET and KET+PRO than in PRO but were not different from each other (*P* = 0.613).

### Plasma glucose, insulin, and amino acid concentrations

**FIGURE 4:**
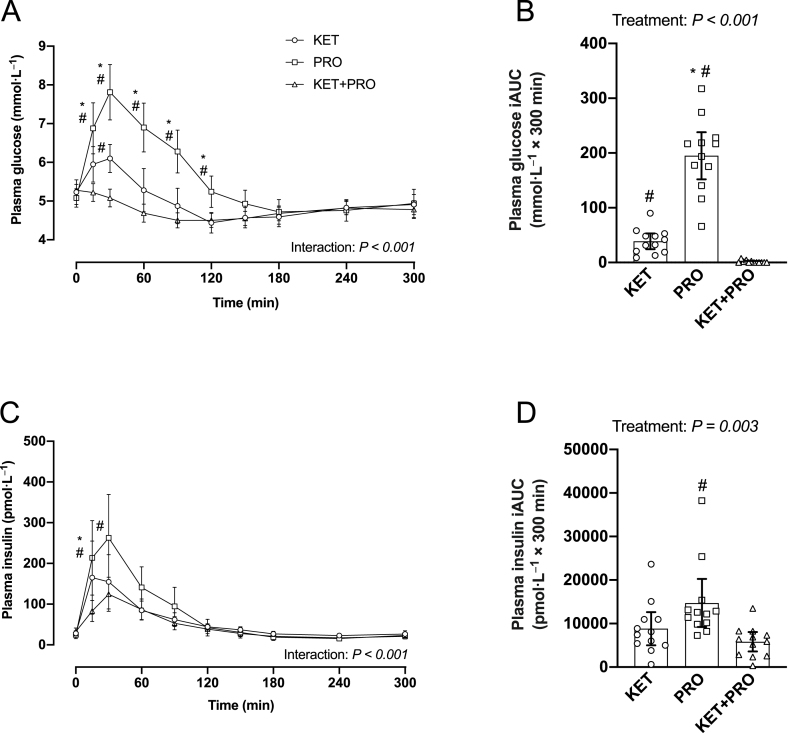
Plasma glucose (A) concentration (mmol/L) and insulin (C) concentration (pmol/L) during basal postabsorptive conditions (*t* = 0 min) and during postprandial conditions (*t* = 15–300 min), and corresponding iAUC for glucose (B) and insulin (D) after beverage intake in young males. Values represent means ± 95% CI and individual participant data, *n* = 12 per treatment. Time-course data were analyzed with a 2-factor repeated-measures ANOVA. Bonferroni post hoc tests were performed to determine the difference between treatments within each time point. iAUC data were analyzed with a 1-factor ANOVA. Bonferroni post hoc tests were performed to detect differences between treatments. Time-course data: ∗a difference from KET within each time point; ^#^a difference from KET+PRO within each time point, *P* < 0.05. The iAUC data: ∗a difference from KET; ^#^a difference from KET+PRO, *P* < 0.05. iAUC, incremental area under the curve; KET, treatment consisting of 0.36 g/kg/body weight of the ketone monoester (R)-3-hydroxybutyl (R)-3-hydroxybutyrate; KET+PRO, treatment consisting of 0.36 g/kg/body weight of the ketone monoester (R)-3-hydroxybutyl (R)-3-hydroxybutyrate co-ingested with 10 g whey protein; PRO, treatment consisting of 10 g whey protein.

Plasma glucose (Figure 4A) concentration (mmol/L) increased (interaction, *P* < 0.001) and was greater in PRO than in both KET and KET+PRO from 15 to 120 min in the postprandial period. At 30 min, plasma glucose concentration was greater in KET (*P* = 0.007) than that in KET+PRO. The iAUC for glucose (Figure 4B) over the 300-min postprandial period was greater (*P* < 0.001) in PRO than that in both KET and KET+PRO. The iAUC for glucose was also greater (*P* < 0.001) in KET than that in KET+PRO. Plasma insulin (Figure 4C) concentration (pmol/L) increased (interaction, *P* < 0.001) and was greater in PRO than that in KET+PRO from 15 to 30 min in the postprandial period and was also greater than that in KET at 15 min. The iAUC for insulin (Figure 4D) over the 300-min postprandial period was greater (*P* = 0.003) in PRO than that in KET+PRO.

**FIGURE 5:**
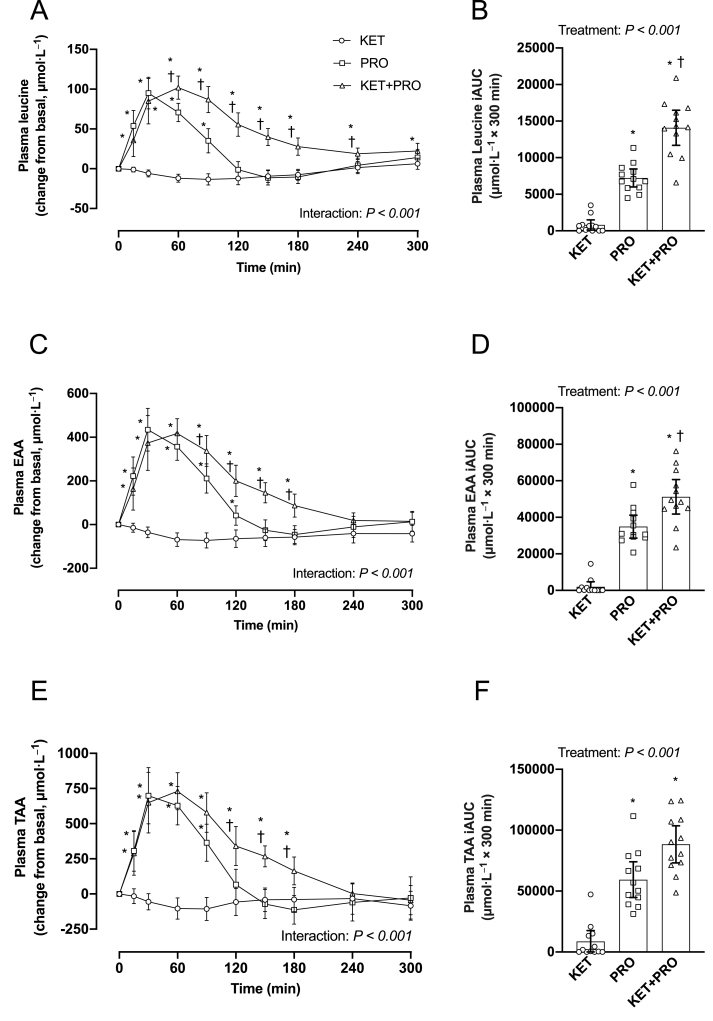
Change in plasma leucine (A), EAA (C), and TAA (E) concentrations (μmol/L) from basal postabsorptive conditions (*t* = 0 min) during postprandial conditions (*t* = 15–300 min), and corresponding iAUC for leucine (B), EAA (D), and TAA (F) after beverage intake in young males. Values represent means ± 95% CI and individual participant data, *n* = 12 per treatment. Time-course data were analyzed with a 2-factor repeated-measures ANOVA. Bonferroni post hoc tests were performed to determine the difference between treatments within each time point. iAUC data were analyzed with a 1-factor ANOVA. Bonferroni post hoc tests were performed to detect differences between treatments. If a significant Levene test was determined, Welch ANOVA, and the Dunnett T3 post hoc comparison were used accordingly to detect differences between treatments. Time-course data: ∗a difference from KET; ^†^a difference from PRO within each time point, *P* < 0.05. The iAUC data: ∗a difference from KET; ^†^a difference from PRO, *P* < 0.05. EAA, essential amino acid; iAUC, incremental area under the curve; KET, treatment consisting of 0.36 g/kg/body weight of the ketone monoester (R)-3-hydroxybutyl (R)-3-hydroxybutyrate; KET+PRO, treatment consisting of 0.36 g/kg/body weight of the ketone monoester (R)-3-hydroxybutyl (R)-3-hydroxybutyrate co-ingested with 10 g whey protein; PRO, treatment consisting of 10 g whey protein; TAA, total amino acid.

Postprandial plasma leucine (Figure 5A) concentration (μmol/L) increased (interaction, *P* < 0.001) and was greater in KET+PRO than that in KET from 15 to 300 min, and was greater compared with PRO from 60 to 240 min. Postprandial plasma leucine concentration was also greater in PRO than that in KET from 15 to 90 min. The iAUC for leucine (Figure 5B) over the 300-min postprandial period was greater (*P* < 0.001) in both KET+PRO and PRO compared with KET. The iAUC for leucine was also greater (*P* < 0.001) in KET+PRO than that in PRO. Postprandial plasma EAA (Figure 5C) concentration (μmol/L) increased (interaction, *P* < 0.001), and was greater in KET+PRO compared with that in KET from 15 to 180 min, and was greater compared with PRO from 90 to 180 min. Postprandial plasma EAA concentration was greater in PRO than that in KET from 15 to 120 min. iAUC for EAA (Figure 5D) over the 300-min postprandial period was greater (*P* < 0.001) in both KET+PRO and PRO than that in KET. The iAUC for EAA was also greater (*P* = 0.016) in KET+PRO than that in PRO. Postprandial plasma total amino acid (TAA) (Figure 5E) concentration (μmol/L) increased (interaction, *P* < 0.001) and was greater in KET+PRO than that in KET from 15 to 180 min and was also greater compared with PRO from 120 to 180 min. Postprandial plasma TAA concentrations were also greater in PRO than that in KET from 15 to 90 min. The iAUC for TAA (Figure 5F) over the 300-min postprandial period was greater (*P* < 0.001) in both KET+PRO and PRO than that in KET but were not different from each other (*P* = 0.056).

### Plasma-free L-[ring-^2^H_5_]-phenylalanine enrichment

**FIGURE 6:**
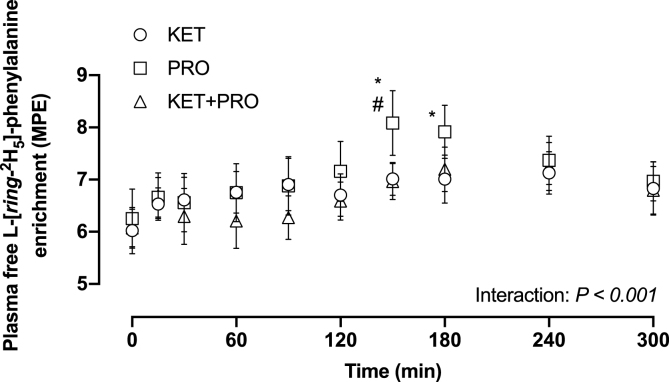
Plasma-free L-[*ring*-^2^H_5_]-phenylalanine enrichment (MPE). Values represent means ± 95% CI, *n* = 12 per treatment. Data were analyzed with a 2-factor repeated-measures ANOVA. Bonferroni post hoc tests were performed to determine the difference between means within each time point. ∗A difference from KET within each time point; ^#^a difference from KET+PRO within each time point, *P* < 0.05. KET, treatment consisting of 0.36 g/kg/body weight of the ketone monoester (R)-3-hydroxybutyl (R)-3-hydroxybutyrate; KET+PRO, treatment consisting of 0.36 g/kg/body weight of the ketone monoester (R)-3-hydroxybutyl (R)-3-hydroxybutyrate co-ingested with 10 g whey protein; MPE, mole percent excess; PRO, treatment consisting of 10 g whey protein.

Before treatment ingestion, mean plasma-free L-[*ring*-^2^H_5_]-phenylalanine (Figure 6) enrichment (MPE) was 6.0 (95% CI: 5.6, 6.4), 6.3 (95% CI: 5.8, 6.8), and 6.1 (95% CI: 5.8, 6.4) in the KET, PRO, and KET+PRO, respectively, with no difference between treatment groups. Plasma-free L-[*ring*-^2^H_5_]-phenylalanine enrichment increased (interaction, *P* < 0.001) and was greater in PRO than that in KET from 150 to 180 min in the postprandial period. Plasma-free L-[*ring*-^2^H_5_]-phenylalanine enrichment was also greater (*P* = 0.002) in PRO than that KET+PRO at 150 min. During the entire 300-min postprandial period, mean plasma L-[*ring*-^2^H_5_]-phenylalanine enrichment was 6.8 (95% CI: 6.5, 7.1), 7.1 (95% CI: 6.7, 7.5), and 6.6 (95% CI: 6.3, 6.9) for KET, PRO, and KET+PRO groups, respectively.

### Myofibrillar FSR

**FIGURE 7:**
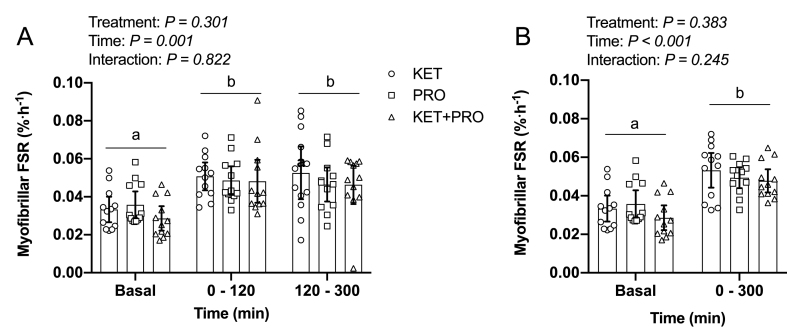
Basal (ie, overnight postabsorptive) and both early (0–120 min) and late (120–300 min) postprandial (A) and basal (ie, overnight postabsorptive) and aggregate (0–300 min) postprandial (B) myofibrillar FSR (%/h). Values are means ± 95% CI and individual participant data, *n* =12 per treatment. Data were analyzed with a 2-factor repeated-measures ANOVA. Times without a common letter differ, *P* < 0.05. FSR, fractional synthesis rate; KET, treatment consisting of 0.36 g/kg/body weight of the ketone monoester (R)-3-hydroxybutyl (R)-3-hydroxybutyrate; KET+PRO, treatment consisting of 0.36 g/kg/body weight of the ketone monoester (R)-3-hydroxybutyl (R)-3-hydroxybutyrate co-ingested with 10 g whey protein; PRO, treatment consisting of 10 g whey protein.

Myofibrillar FSR during both the early (0–120 min) and late (120–300 min) phase of the postprandial period (Figure 7A) was significantly greater (time: *P* = 0.001) than that in basal postabsorptive conditions (−180 to 0 min), with no differences between treatment groups (treatment: *P* = 0.301) or treatment × time interaction (interaction: *P* = 0.822). Similarly, myofibrillar FSR assessed during the aggregate (0–300 min) postprandial period (Figure 7B) was significantly greater (Time: *P* < 0.001) than that in basal postabsorptive conditions, with no difference between treatment groups (treatment: *P* = 0.383) or treatment × time interaction (interaction: *P* = 0.245). In particular, KET, PRO, and KET+PRO stimulated aggregate (0–300 min) postprandial MyoPS rates higher than basal conditions (absolute change) by: 0.020%/h (95% CI: 0.013, 0.027%/h), 0.014%/h (95% CI: 0.009, 0.019%/h), and 0.019%/h (95% CI: 0.014, 0.024%/h), respectively.

### Intramuscular signaling

**FIGURE 8:**
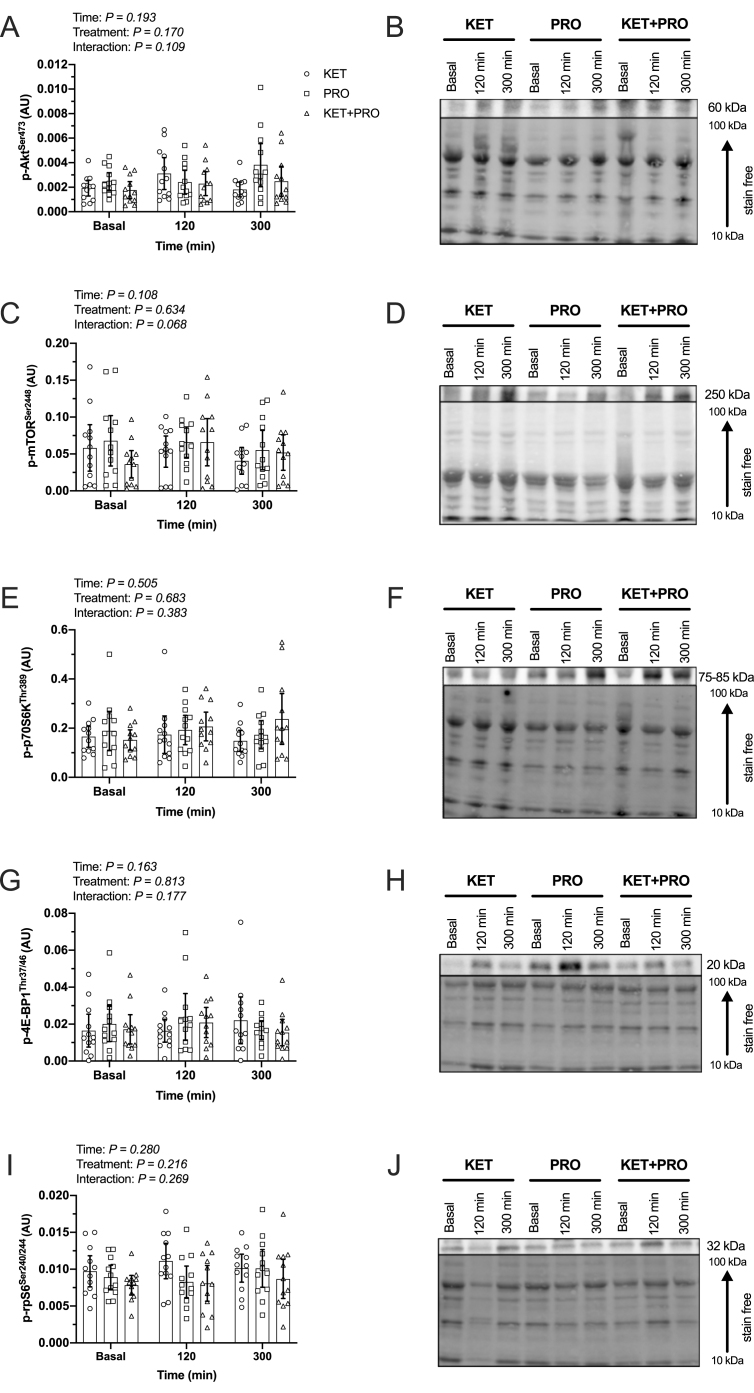
Phosphorylation status of p-Akt^Ser473^ (A), p-mTOR^Ser2448^ (C), p-p70S6K^Thr389^ (E), p-4E-BP1^Thr37/46^ (G), and p-rpS6^Ser240/244^ (I) during basal (ie, overnight postabsorptive) conditions and at 120 and 300 min during postprandial conditions after beverage intake in young males. Representative Western blot images and loading control for the phosphorylation status of p-Akt^Ser473^ (B), p-mTOR^Ser2448^ (D), p-p70S6K^Thr389^ (F), p-4E-BP1^Thr37/46^ (H), and p-rpS6^Ser240/244^ (J) during basal conditions (*t* = 0 min) and at 120 and 300 min during postprandial conditions after beverage intake in young males. Values represent means ± 95% CI and individual participant data, *n* = 12 per treatment. Data were analyzed with 2-factor repeated-measures ANOVA. 4E-BP1, eukaryotic translation initiation factor 4E-binding protein 1; Akt, protein kinase B; AU, arbitrary unit; KET, treatment consisting of 0.36 g/kg/body weight of the ketone monoester (R)-3-hydroxybutyl (R)-3-hydroxybutyrate; KET+PRO, treatment consisting of 0.36 g/kg/body weight of the ketone monoester (R)-3-hydroxybutyl (R)-3-hydroxybutyrate co-ingested with 10 g whey protein; mTOR, mechanistic target of rapamycin; p70S6K, 70 kDa ribosomal protein S6 kinase; PRO, treatment consisting of 10 g whey protein; rpS6, ribosomal protein S6.

The phosphorylation status of Akt^Ser473^ (Figure 8A,B), mTOR^Ser2448^ (Figure 8C,D), p70S6K^Thr389^ (Figure 8E,F), 4E-BP1^Thr37/46^ (Figure 8G,H), and rpS6^Ser240/244^ (Figure 8I,J) did not differ over time (Time, all *P* > 0.05) or between nutritional treatments (treatment, all *P* > 0.05).

## Discussion

In this study, we evaluated postprandial changes in capillary blood β-OHB, plasma glucose, insulin, and amino acid concentrations, MyoPS rates, and the phosphorylation status of select protein targets within the Akt-mTORC1 signaling cascade in response to acute ingestion of a ketone monoester (KET), 10 g whey protein (PRO), or their co-ingestion (KET+PRO) administered in the overnight postabsorptive state in healthy young males. KET and KET+PRO elevated capillary blood β-OHB concentration (β-OHB C_max_ >3.0 mmol/L) compared with PRO. Both PRO and KET+PRO increased postprandial plasma amino acid concentration; however, both the temporal and iAUC response differed between treatments. Leucine, EAA, and TAA iAUC over the 300-min postprandial period was greater in both PRO and KET+PRO than that in KET. In addition, leucine and EAA iAUC was greater in KET+PRO than that in PRO. KET, PRO, and KET+PRO ingestion increased postprandial MyoPS rates higher than basal postabsorptive rates with no difference between treatments. There was no discernable difference among treatments in the phosphorylation status of mTORC1-related signaling targets at the time points examined.

Consistent with work from our laboratory [4] and others [3,9], acute oral ingestion of the ketone monoester (R)-3-hydroxybutyl (R)-3-hydroxybutyrate resulted in a pronounced (β-OHB C_max_ > 3.0 mmol/L), rapid (within ∼30 min), but transient (∼180 min) nutritional ketosis (Figure 3A). In this study, a small amount of carbohydrate (12.2 g) was added to KET to match the energy provided from 10 g whey protein in KET+PRO. Both treatments induced a similar pronounced, rapid, but transient rise in blood β-OHB concentration, consistent with findings from other studies using a similar dose of the same ketone monoester [3,4]. This confirms that exogenous ketone monoester drinks represent an efficacious way to induce ketosis.

Intravenous ketone body infusions have been reported to stimulate pancreatic β-cell insulin secretion [[21], [22], [23]]. Similarly, studies using exogenous ketone monoester drinks have reported an increase in blood insulin concentration in the fasted state [24,25] and during a postexercise hyperglycemic clamp [26]. The greater increase in plasma insulin concentration in PRO compared with KET and KET+PRO at 15 min in the postprandial period is likely due to the greater amount of total carbohydrate ingested (Table 1). Although insulin is a key peptide hormone involved in the regulation of muscle protein turnover, a systematic review and meta-analysis concluded its primary role is to suppress muscle protein breakdown rather than stimulate MPS rates [27]. For example, Greenhaff et al. [28] reported that MPS rates were identical in response to an EAA infusion whether insulin was “clamped” at basal postabsorptive (5 mU/L) or physiologically high (167 mU/L) concentration. Therefore, in this study subtle differences in insulin concentration between treatments are unlikely to have influenced postprandial MyoPS rates.

The postprandial increase in circulating EAA and leucine in particular, after protein ingestion, is a key regulator of MPS rates [[29], [30], [31], [32]]. Although previous studies have examined the effects of protein co-ingestion with carbohydrate [[33], [34], [35]] or fat [36,37] on postprandial plasma aminoacidemia, this study is the first, to our knowledge, to investigate the effects of ketone monoester co-ingestion with protein on postprandial plasma amino acid concentrations. As expected, there was an increase in postprandial plasma leucine concentration (Figure 5A,B) after both PRO and KET+PRO; however, KET+PRO resulted in a more protracted leucinemia and greater iAUC than that in PRO. Similar findings were observed for plasma EAA (Figure 5C,D) and TAA (Figure 5E,F) concentration. The reason(s) for differences in postprandial plasma amino acid concentrations between PRO and KET+PRO is unclear, but may relate to differences in plasma insulin concentration in PRO compared with KET+PRO (Figure 4C,D). Previous studies [38,39] have reported that increases in plasma insulin concentration can reduce plasma amino acid concentration. Insulin plays a key role in facilitating increases in microvascular perfusion [40] and supporting amino acid delivery/uptake into skeletal muscle [41]. Therefore, the attenuated increase in postprandial plasma amino acid concentration that coincided with a greater increase in circulating insulin concentration may suggest that disappearance of amino acids from the circulation into skeletal muscle was enhanced in PRO than that in KET+PRO. Alternatively, KET+PRO may have decreased amino acid retention in splanchnic tissues compared with PRO, allowing more of the ingested amino acids to become available in the circulation. Future studies assessing protein digestion/absorption kinetics could address this question.

Ketone bodies have long been implicated in the regulation of protein metabolism during prolonged fasting and starvation [42,43]. During prolonged glucose deprivation, ketone bodies replace glucose as the primary fuel for the brain, supplying >50% of the brain’s energy [44]. Starvation-induced ketosis may reduce the requirement for protein (muscle) catabolism to provide gluconeogenic amino acid precursors [5,45,46]. In support of this notion, hyperketonemia is associated with improved nitrogen balance in traumatized man [47] and postoperative patients [48]. Furthermore, infusion of sodium DL-β-OHB reduces plasma alanine concentration and urinary nitrogen excretion in response to prolonged fasting [5]. More recent research has demonstrated that β-OHB has potent anti-proteolytic effects in muscle during acute inflammatory stress [49] and under catabolic conditions characterized by combined systemic inflammation, fasting, and bed rest [50]. There is also evidence that β-OHB can stimulate MPS rates [8,9]. For example, Nair et al. [8] reported that an 8-h intravenous infusion of sodium DL-β-OHB (12.5 μmol/kg/min) to a plasma β-OHB of ∼2 mM, reduced leucine oxidation by ∼30%, and stimulated mixed MPS rates by ∼10% in healthy young adults. In this study, KET, PRO, and KET+PRO elicited a similar increase in postprandial MyoPS rates (Figure 7A,B), despite no exogenous source of amino acids in KET, and pronounced differences in the change in postprandial plasma leucine and EAA concentration in PRO and KET+PRO. The postprandial increase in MyoPS rates over 0–300 min (∼36%) in PRO is similar to that of other studies examining the effects of a 10-g dose of whey protein in resting muscle (∼22%–24%) [11,18]. Although leucine and EAA are key nutrient regulators of MPS, differences in the pattern of postprandial plasma amino acid concentration do not always translate to differences in MPS rates [[51], [52], [53], [54]]. Furthermore, MPS rates can be stimulated in the absence of exogenous leucine or EAA using the leucine metabolite β-hydroxy-β-methylbutyrate [55] and amino acid citrulline [56]. The stimulation of MyoPS rates in KET must have been supported using EAA from other pools (eg, plasma, intracellular, or protein through tissue protein breakdown). Although ketone bodies have been reported to potentiate protein synthesis in vitro in low-dose (1.5 mM) leucine-stimulated myotubes [9], we observed no additional stimulatory effect of KET+PRO on MyoPS rates. This suggests that amino acid availability and/or nutrient signals that serve to stimulate MyoPS were similar in all treatments.

Signaling via the mTORC1 pathway did not differ between basal and postprandial conditions regardless of treatment (Figure 8A–J). It is possible that the timing of biopsy sample collection (120 and 300 min) may have missed the “window” to capture changes in anabolic signaling events. Typically, signaling responses peak within 1–2 h of feeding [[57], [58], [59]] and, so, any signaling events that occurred <2 h posttreatment may have been missed [60]. Alternatively, discordance between MPS rates and anabolic signaling events in response to nutritional stimulation in human muscle has been previously reported [28]. In contrast to this study, Vandoorne et al. [9] reported that ketone monoester co-ingestion with a high-dose protein–carbohydrate drink throughout (every 30 min) 300 min of postexercise recovery enhanced the phosphorylation status of 4E-BP1%γ and S6K1^Thr389^ at 300 min compared with the same protein–carbohydrate drink with long-chain triglycerides. Differences between this study and that of Vandoorne et al. [9] may relate to the exercise stimulus and greater dose of ingested protein/leucine (leucine intake: ∼12.1 ± 0.4 g per participant). The specific molecular mechanisms by which ketone bodies regulate MyoPS in humans requires additional research.

There are some potential limitations to this study that warrant acknowledgment. First, we did not include a negative control (eg, flavored water) with which to compare KET, PRO, and KET+PRO treatments. Nonetheless, all treatments stimulated postprandial MyoPS rates higher than basal rates. Second, although carbohydrate was added to both KET and PRO treatments to better match the energy of the KET+PRO treatment, this may have augmented blood glucose and insulin responses, and thereby plasma amino acid concentration. However, given insulin per se does not stimulate MPS rates in humans [27], subtle differences in insulin concentration between treatments are unlikely to have influenced the postprandial MyoPS response. Third, to evaluate their effects on the selected outcome measures, nutritional treatments were administered in the overnight postabsorptive state. However, meal intake before ingestion of a ketone monoester drink has been reported [24] to reduce blood β-OHB C_max_ by ∼33% compared with ingestion of the same ketone monoester drink on an empty stomach (β-OHB: 3.3 compared with 2.2 mmol/L). Therefore, whether the effects reported in this study would also occur after ketone monoester administration in the fed state is unclear. Finally, only young males were studied because our primary outcome measure (MyoPS rates) may have been confounded by mixed-sex and/or age-related responses to ketone monoester intake. Research exploring the effects of ketone bodies on protein metabolism in females and in older adults who may display anabolic resistance to nutritional stimuli is needed.

In conclusion, we demonstrate that acute intake of a ketone monoester, with or without dietary protein co-ingestion, results in elevated β-OHB/hyperketonemia. Ketone monoester co-ingestion with dietary protein augments the increase in postprandial plasma leucine, EAA, and TAA concentration compared with dietary protein without ketone monoester co-ingestion. Despite differences in postprandial plasma aminoacidemia, acute ingestion of a ketone monoester (0.36 g/kg/body weight) eliciting a β-OHB C_max_ of ∼3.2 mmol/L, 10 g whey protein, or their co-ingestion in the overnight postabsorptive state elicit similar increases in postprandial MyoPS rates in vivo in healthy young males.

## Acknowledgments

We thank Marie Lamarche (Research Institute of the McGill University Health Centre, Montréal Quebec, Canada) for her assistance with sample preparation for analyses through ultraperformance liquid chromatography–mass spectrometry. We thank Dr. Stéphanie Chevalier (School of Human Nutrition, McGill University Montréal, Quebec, Canada) for helpful discussion throughout this project.

## Author contributions

The authors’ responsibilities were as follows – JL, TAC-V: designed the research; SJH, JL, SEH, DM, MD, AG, JAM, TAC-V: conducted the research; JAM, GG, TAC-V: provided essential materials; SJH, JL, MD, AG, GG, TAC-V: analyzed data; SJH, JL: performed the statistical analysis; SJH, JL, TAC-V: wrote the manuscript; TAC-V: had primary responsibility for final content; and all authors: have read and approved the final manuscript.

### Conflict of interest

The authors declare no conflicts of interest.

### Funding

McGill Sylvan Adams Sports Science Institute Award and Natural Sciences and Engineering Research Council of Canada Award was awarded to TAC-V.

## Data Availability

Data described in the manuscript will be made available on request pending application to and approval from the corresponding author.
