# Supplementary material for: Acute ingestion of a ketone monoester, whey protein, or their co-ingestion in the overnight postabsorptive state elicit a similar stimulation of myofibrillar protein synthesis rates in young males: a double-blind randomized trial
Source: Am J Clin Nutr. 2024 Jan 11;119(3):716–29. doi: 10.1016/j.ajcnut.2024.01.004 (PMC10972741; doi:10.1016/j.ajcnut.2024.01.004)
Supplement: Multimedia component 1 [file mmc1.docx]

**Acute ingestion of a ketone monoester, whey protein, or their co-ingestion in the overnight postabsorptive state elicit a similar stimulation of myofibrillar protein synthesis rates in young males: a double-blind randomized trial**

*Sarkis J. Hannaian^1,2^, Jamie Lov^1^, Stephanie E. Hawley^1^, Manon Dargegen^2^, Divine Malenda^1^, Ari Gritsas^2^, Gilles Gouspillou^3^, José A. Morais^1,2,4^, Tyler A. Churchward-Venne^1,2,4^*

^1^Department of Kinesiology and Physical Education, McGill University, Montreal, QC H2W 1S4, Canada; ^2^Research Institute of the McGill University Health Centre, Montreal, QC H4A 3J1, Canada; ^3^Département des Sciences de l’activité Physique, Faculté des Sciences, UQAM, Montréal, QC H2X 1Y4, Canada; ^4^Division of Geriatric Medicine, McGill University, Montreal, QC H3T 1E2, Canada

**ONLINE SUPPLEMENTARY MATERIAL: SUPPLEMENTAL METHODS**

*Plasma glucose, insulin, and amino acid analyses*

Plasma glucose and insulin concentrations were measured at the Clinical Biochemistry Laboratory of the McGill University Health centre (Montreal, QC). Plasma glucose concentration was measured using a chemistry analyzer (Beckman Coulter AU5800), while insulin was measured via immunoenzymatic assay (Beckman Dx1800). According to data provided by the laboratory, the coefficient of variation (CV) was 2.5% for glucose and 5% for insulin.

Plasma amino acid concentration was assessed at the Proteomics and Clinical Mass Spectrometry platform at the Research Institute of the McGill University Health Centre (Montreal, QC). Amino acids were extracted from plasma using protein precipitation and derivatized with 6- aminoquinolyl-N-hydroxysuccinimidyl carbamate (AQC; Toronto Research Chemicals; ON, Canada) for analysis using reversed phase ultra-performance liquid chromatography mass spectrometry (UPLC-MS). Plasma samples were extracted alongside a calibration curve of amino acids in 0.1N HCl with norvaline as an internal standard (all amino acids and norvaline purchased from Sigma-Aldrich; MO, USA). A calibration curve of 5 to 1000 μM was used for all amino acids except cysteine (2.5 to 500 μM). An internal standard working solution (ISWS) containing 50 μM norvaline in 5% 5-sulfosalicylic acid was used to extract plasma and calibration samples. ISWS aliquots (25 μL) were added to sample aliquots (25 μL) in microcentrifuge tubes, vortexed and centrifuged at 10,000 × g at 10°C for 10 mins. Supernatant aliquots (10 μL) were transferred into glass tubes containing 70 μL buffer solution (0.2M sodium borate pH 8.8) along with 20 μL derivatization solution (10mM AQC in acetonitrile), mixed and incubated for 10 min at 55°C. After cooling to room temperature, aliquots (10 μL) were transferred to autosampler vials containing 990 μL Type-1 water for UPLC-MS analysis. Extracts were analyzed by UPLC-MS using an Agilent 6460 triple quadrupole mass spectrometer coupled with an Agilent 1290 UPLC system (Agilent; CA, USA). Extracts (5 μL) were injected onto an Agilent Eclipse Plus C18 100 x 2.1 mm (1.8 μm) column and chromatographed with a reverse phase gradient at 0.200 mL/min using 0.1% formic acid in water and 0.1% formic acid in acetonitrile. The derivatized amino acids were detected using electrospray positive mode ionization followed by MS/MS fragmentation. Data acquisition was performed using Agilent MassHunter Data Acquisition (version B.04.01) software. Peak area measurements from selected product ions, calibration curve regression analysis and resulting sample quantification were performed using Agilent MassHunter Quantitative Analysis (version B.05.00) software.

*Blood and muscle phenylalanine enrichment analyses*

Plasma free, plasma protein-bound, and muscle protein-bound L-[*ring*-^2^H_5_]-phenylalanine enrichments were assessed using separate sample preparation methods but with the same UPLC-MS method. Plasma free L-[*ring*-^2^H_5_]-phenylalanine enrichments were determined using protein precipitation. Aliquots (25 μL) of an ISWS containing 50 μM methyl-DL-phenylalanine in 5% 5-sulfosalicylic acid were added to sample aliquots (25 μL) in microcentrifuge tubes, vortexed, and centrifuged at 10,000 × g at 4°C for 5 min. Supernatant aliquots (25 μL) were transferred to 75 μL Type-1 water in another set of microcentrifuge tubes and vortexed. Aliquots (10 μL) were transferred to autosampler vials containing 490 μL Type-1 water for UPLC-MS analysis.

Protein-bound plasma L-[*ring*-^2^H_5_]-phenylalanine enrichments were determined using protein isolation followed by acid hydrolysis and solid phase extraction. Plasma sample aliquots (250 μL) were added to 10% trichloroacetic acid in water aliquots (250 μL) in microcentrifuge tubes, vortexed, and centrifuged at 1,000 × g at 4°C for 10 min. The supernatants were discarded and 850 µL aliquots of 10% trichloroacetic acid in water were added. The pellets were resuspended with vigorous mixing and centrifuged again at 1,000 × g at 4°C for 10 min. The supernatant removal with pellet resuspension and centrifugation were repeated twice more. All possible supernatant was removed from the final pellets, and they were resuspended in 800 µL 6N HCl for acid hydrolysis. The resuspended pellets were transferred to screw cap glass tubes. The microcentrifuge tubes were rinsed twice with 800 µL 6N HCl and the solutions transferred to their corresponding glass tubes for a final hydrolysis solution volume of 2.4 mL. The samples were heated in a dry bath at 105°C for 16.5 hours. The hydrolysates were cooled to room temperature and transferred to glass tubes. Aliquots (1 mL) of hydrolysates were added to pre-conditioned (1 mL methanol then 1 mL 0.1N HCl) 30 mg Strata-X-C cartridges (Phenomenex; CA, USA). The cartridges were then washed twice with 1 mL water. The analytes were then eluted with two 600 µL aliquots of 5% ammonium hydroxide in water. The eluted samples were placed to dry and evaporate under nitrogen at 37°C and reconstituted in 1 mL water.

To determine muscle (myofibrillar) protein-bound enrichments, a piece (∼30 mg) of muscle was homogenized on ice using a standing tissue homogenizer (Kinematica Inc.; USA) in 2mL Optima water (Fisher Scientific; ON, Canada). The homogenate was transferred to screw top conical tubes, vortexed, and centrifuged at 1,500 × g at 4°C for 10 mins to separate the myofibrillar and collagen proteins. The myofibrillar pellet was broken up and washed with 2 mL Optima water. The sample was then vortexed and centrifuged at 1,500 × g at 4°C for 10 mins after which the supernatant was aspirated and the washing process was repeated twice more using the same steps. After the washes, the myofibrillar pellet was resolubilized in 1.5ml 0.3M NaOH (Fisher Scientific; ON, Canada), vortexed, and centrifuged at 1,500 × g at 4°C for 10 mins. The resulting supernatant was hydrolyzed with 1.5 mL 6M HCL (Fisher Scientific; ON, Canada) inside a reaction vial. The reaction vial was heated to 110°C for 16 h. Subsequently, aliquots (0.4 mL) were transferred to glass culture tubes and evaporated under nitrogen gas at 37°C until completely dry and then stored at -20°C. The dried myofibrillar fractions were reconstituted with 1 mL Type-1 water. Aliquots (50 µL) were transferred to injection vials containing 950 µL internal standard solution (10 nM methyl-DL-phenylalanine in water) for UPLC-MS analysis.

Enriched samples were analyzed by UPLC-MS using the same Agilent 6460 triple quadrupole mass spectrometer coupled with an Agilent 1290 UPLC system with the same Agilent MassHunter Data Acquisition and Quantitative Analysis software. Extracts (5 μL) were injected onto an Agilent Eclipse Plus C18 50 x 2.1 mm (1.8 μm) column and chromatographed with a reverse phase gradient at 0.300 mL/min using 0.1% formic acid in water and 0.1% formic acid in acetonitrile. L-phenylalanine, L-[*ring*-^2^H_5_]-phenylalanine, and methyl-DL-phenylalanine were detected using electrospray positive mode ionization followed by MS/MS fragmentation. L-phenylalanine was monitored using three transitions at *m/z* 166-120 (primary isotope), *m/z* 168-120 (+2 isotope) and *m/z* 169-120 (+3 isotope). L-[*ring*-^2^H_5_]-phenylalanine was monitored at *m/z* 171-125 (primary isotope). Methyl-DL-phenylalanine (internal standard) was monitored at *m/z* 180-134 (primary isotope). The internal standard was added to monitor system stability and was not used in any enrichment calculations. Additional isotopes for L-phenylalanine were monitored to verify potential signal saturation in samples with high L-phenylalanine amounts. Tracer-to-tracee calculations for free plasma samples were made by dividing L-[*ring*-^2^H_5_]-phenylalanine peak area by L-phenylalanine peak area and multiplying by a response factor. The response factor was used to correct for the difference in UPLC-MS response between labelled and unlabelled phenylalanine. A tracer-tracee ratio (TTR) calibration curve was deemed unnecessary to use for free plasma as the L-[*ring*-^2^H_5_]-phenylalanine amounts were large in the samples and well above the limit of detection.

Low L-[*ring*-^2^H_5_]-phenylalanine signals were expected so tracer-to-tracee calculations for bound muscle samples were made by using a TTR solution calibration curve which was injected along with the samples. The curve consisted of standards with different amounts of L-[*ring*-^2^H_5_]-phenylalanine (0.5-10 nM) with a constant amount of phenylalanine (5000 nM) which resulted in a TTR curve range from 0.0001 to 0.0020 (0.01% to 0.20%). No significant differences were observed in determined TTR values using L-phenylalanine concetrations from 1000 to 5000 nM which encompassed the range observed in the sample extracts.

*Western Blotting*

A portion of skeletal muscle extracted during the biopsy procedure (~15-30 mg) was used for Western blotting. Muscle tissue was homogenized in 10 volumes of an extraction buffer composed of tris base 50mM, NaCl 150mM, triton X-100 1%, sodium deoxycolate 0.5%, SDS 0.1% and 10μl/mL of a protease and phosphatase inhibitor cocktail (A32959, Thermofisher). The homogenates were centrifuged at 12,000 g for 15 mins at 4°C. Protein content in the supernatant was determined using the Bradford method. Aliquots of supernatant were mixed with Laemmli buffer 4x (Biorad) containing β-mercaptoethanol, and subsequently boiled at 95°C for 5 min. Approximately 20 μg of protein were loaded into gradient (4-15%) – except for Phospho-mTOR that was loaded into gradient (7.5%) – and stain-free gels (Mini PROTEAN® TGX Stain-Free TM Gels, Biorad), electrophoresed by SDS-PAGE and then transferred to polyvinylidene fluoride membranes (PVDF, Biorad). A stain-free blot image was taken using the ChemiDocTM Touch Imaging System for total protein measurement in each sample lane. Membranes were blocked in tris-buffered saline + 1% Tween® 20 (TBS-T) + 5% bovine serum albumin (BSA) for 1 hour at room temperature and then incubated with the specific primary antibodies for 1 hour 30. The complete list of antibodies and dilutions used for immunoblotting analyses can be found in **Table 2***.* All antibodies were diluted in blocking buffer. Membranes were washed in TBS-T (3×5 min) and incubated with HRP-conjugated secondary goat anti-rabbit IgG (Abcam, cat# Ab6721, 1/10,000) or goat anti-mouse IgG (Abcam, cat# Ab6728, 1/10,000) secondary antibodies for 1 hour at room temperature, before further washing in TBS-T (3×5 min). Signals were detected using enhanced chemiluminescence substrate (Biorad, Clarity ECL substrate, 170-5061 or Thermo Fisher, West Femto, 34094) and the ChemiDocTM Touch Imaging System. Bands were analyzed using the ImageLab 6.1 software (Biorad). For each sample, the ECL signal for the protein of interest was normalized to the intensity of the stain-free blot image of the corresponding sample (i.e., the intensity of the stain-free blot image was used as a loading control).
